# A systematic review of left ventricular cardio-endoscopic surgery

**DOI:** 10.1186/s13019-017-0599-z

**Published:** 2017-05-25

**Authors:** Erdinc Soylu, Emaddin Kidher, Hutan Ashrafian, George Stavridis, Leanne Harling, Thanos Athanasiou

**Affiliations:** 1 Department of Surgery and Cancer, Imperial College London, 10th Floor, QEQM Building, St Mary’s Hospital Campus, South Wharf Road, London, W2 1NY UK; 2 Department of Cardiac Surgery, Onassis Cardiac Surgery Centre, Athens, Greece

**Keywords:** Cardioscopy, Left ventricular tumor, Thrombus, Hypertrophic myocardium

## Abstract

Better visualisation, accurate resection and avoidance of ventriculotomy associated with use of endoscopic devices during intracardiac surgery has led to increasing interest in their use. The possibility of combining a cardio-endoscopic technique with either minimally invasive or totally endoscopic cardiac surgery provides an incentive for its further development. Several devices have been used, however their uptake has been limited due to uncertainty around their impact on patient outcomes. A systematic review of the literature identified 34 studies, incorporating 54 subjects undergoing treatment of left ventricular tumours, thrombus or hypertrophic myocardium using a cardio-endoscopic technique. There were no mortalities (0%; 0/47). In 12 studies, the follow-up period was longer than 30 days. There were no post-operative complications apart from one case of atrial fibrillation (2.2%; 1/46). Complete resection of left ventricular lesion was achieved in all cases (100%; 50/50). These successful results demonstrate that the cardio-endoscopic technique is a useful adjunct in resection of left ventricular tumours, thrombus and hypertrophic myocardium. This approach facilitates accurate resection of pathological tissue from left ventricle whilst avoiding exposure related valvular damage and adverse effects associated with ventriculotomy. Future research should focus on designing adequately powered comparative randomised trials focusing on major cardiac and cerebrovascular morbidity outcomes in both the short and long-term. In this way, we may have a more comprehensive picture of both the safety and efficacy of this technique and determine whether such devices could be safely adopted for routine use in minimal access or robotic intra-cardiac surgery.

**Electronic supplementary material:**

The online version of this article (doi:10.1186/s13019-017-0599-z) contains supplementary material, which is available to authorized users.

## Background

The treatment of left ventricular lesions is traditionally performed under direct vision and accessed through left atrium, aorta or ventriculotomy [1–3]. Lesions located in the LV inflow tract are usually accessed through the left atrium and those in outflow tact via the aorta [4–6]. Some are however located deep in the LV cavity close to the apex, where access is difficult without ventriculotomy [7]. Access through the aorta and left atrium may risk damage to valvular structures, and limited vision through these structures may result in incomplete excision of LV lesions. Similarly, ventriculotomy confers a risk of bleeding, conduction dysfunction, impairment of myocardial blood flow, impairment of left ventricular function and aneurysm formation [5, 6, 8, 9].

Use of endoscopic devices to visualise intra-cardiac structures and to assist in excision of left ventricular lesions is a viable alternative [6, 7], which may provide improved visualisation of intracardiac structures and ensure safe, complete resection whilst avoiding the adverse risks associated with ventriculotomy [10, 11]. In addition, this technique has the added benefit of being compatible with use in minimally invasive procedures, further minimising the surgical trauma and associated morbidity [6, 12]. Its use may also be of particular benefit for patients with pre-existing ventricular impairment who would otherwise require ventriculotomy [13–15].

Endoscopes were first used in cardiac surgery to facilitate the assessment and management of benign and malignant pericardial effusions by pericardioscopy [16]. This was later followed by their application to division of vascular rings, closure of patent ductus arteriosus and ventricular septal defects [12, 17, 18]. However, these early studies reported use of rigid endoscopes. In recent years, flexible endoscopes have been introduced and both endoscopic (e.g., forceps, graspers, scissors) and open surgical instruments may now be used as adjuncts to facilitate more complex procedures.

The purpose of this study is to provide a quantitative summary of the evidence related to the use of cardio-endoscopic devices in humans. Our primary aim is to evaluate the effect of the technique on successful treatment of left ventricular lesions. Secondarily we consider the impact this technology has on mortality and post-operative morbidities both in the early and late post-operative period.

## Literature search criteria

### Literature search criteria

An electronic search was performed using MEDLINE, Ovid, EMBASE, Cochrane and the UK National Library for Health databases using the following MeSH terms: “cardioscopic” OR “cardioscopy” OR “endoscopy” OR “endoscopic” OR “scope” OR “thoracoscopic” OR “videotape recording” OR “videotape” AND “recording” OR “video” AND assisted OR “video” AND “heart” OR “heart” OR “cardiac” OR “heart ventricles” OR “heart” AND “ventricles” OR “ventricle” OR “myocardium” OR “myocardial” OR “ventricular” OR valve AND resection OR removal OR excision AND “thrombosis” OR “thrombus” OR “tumour” OR “neoplasms” OR “tumor” OR fibroelastoma OR “myxoma” OR clot AND “humans” AND English AND “adult”. Articles were also identified using the ‘related articles’ function in MEDLINE and screening of the reference lists of manuscripts identified in the original search.

All articles reporting cardio-endoscopic methods in the treatment of left ventricular pathologies including tumour, thrombus and hypertrophic myocardium were included. Owing to the small number of studies, those studies reporting concomitant procedures were not excluded but are discussed in our overall interpretation of results. Studies were excluded if they reported solely results from procedures involving atrium, mitral valve, congenital heart surgery, animals, in vitro testing or children (≤18 years). In addition studies reporting solely on diagnostic use of the cardioscopy or published in languages other than English were also excluded. In the case of duplicate publication, only the most recent study was included.

Data extraction was performed according to a predefined protocol by two independent reviewers (E.S. and E.K.). Short- and long-term outcomes were assessed. Short-term outcomes included: (i) Immediate post-operative mortality (ii) Post-operative major cardiac and cerebrovascular events (MACCE), haemorrhage and other clinical morbidity. Long-term outcomes included: (i) Mortality at last follow-up. The last search date was 01 February 2016.

## Results

### Description of studies

A total of 34 studies [1–15, 19–37] incorporating 54 patients reported outcomes for cardio-endoscopic treatments of left ventricular tumours, thrombus and hypertrophic myocardium (Additional file 1: Table S1; Table 1). A breakdown of the indications for surgery, incision types, and location of the operated pathology is shown in Table 1. The post-operative mortality, morbidity and technique efficacy are shown in Table 2. There were no comparative studies, with all except one [11] were case reports [1–10, 12–15, 19–37].

**Table 1:** Overall patient demographics, pathology and operative technique

Characteristics	Number of studies	Number of cases	Percent (n/total)
Patients	34	54	-
Male: female	32	22:10	-
Mean age (range)	32	-	52.7 (17–82)
Indication			
Thrombus	13	16	29.6% (16/54)
Tumour	21	22	40.7% (22/54)
Hypertrophic and fibrous tissue	2	16	29.6% (16/54)
Tumors Histopathology			
Papillary fibroelastoma	13	14	63.6% (14/22)
Myxoma	6	6	27.3% (6/22)
Benign hemangioma	1	1	4.5% (1/22)
Metastatic synovial sarcoma	1	1	4.5% (1/22)
Location of the lesion within left ventricle			
Body	19	33	61.1% (33/54)
Apex	10	10	18.5% (10/54)
Papillary muscle	3	3	5.5% (3/54)
Mitral valve chord	2	2	3.7% (2/54)
Unreported	1	6	11.1% (6/54)
Number of lesions per case			
One	27	27	50% (27/54)
Two	4	4	7.4% (4/54)
Multiple	1	1	1.9% (1/54)
Unreported	4	22	40.7% (22/54)
As part of another procedure	34	54	7.4% (4/54)
CABG	4	4	100% (4/4)
Incision			
Median sternotomy	23	43	79.6% (43/54)
Right mini thoracotomy	4	4	7.4% (4/54)
Right thoracoscopy	1	1	1.9% (1/54)
Unreported	6	6	11.1% (6/54)
Entry site in to heart			
Aortotomy	22	42	77.8% (42/54)
Left atriotomy	11	11	20.4% (11/54)
Unreported	1	1	1.9% (1/54)
Cross-clamp time (mean)	10	10	59.5 min
Cardiopulmonary bypass time (mean)	8	8	105.3 min

**Table 2:** Mortality, Morbidity and Technique Efficacy

Study	n	Successful removal	Complications (MI, stroke, hemorrhage and other morbidity)	Mortality (At follow-up)	Postoperative follow-up
Duarte et al., [19]	1	100% (1/1)	0% (0/1)	0% (0/1)	4 days
Mazza et al., [12]	1	100% (1/1)	0% (0/1)	0% (0/1)	1 year
Tsukube et al., [15]	1	100% (1/1)	0% (0/1)	0% (0/1)	14 days
Reuthebuch et al., [11]	21	100% (21/21)	0% (0/21)	0% (0/21)	Inpatient duration
Junemann-Ramirez et al., [13]	1	100% (1/1)	0% (0/1)	0% (0/1)	Inpatient duration
Oumeiri et al., [20]	1	--	--	--	--
Kawamoto et al., [21]	1	100% (1/1)	0% (0/1)	0% (0/1)	Inpatient duration
Porcu et al., [22]	1	100% (1/1)	0% (0/1)	0% (0/1)	8 days
Kikuchi et al., [23]	1	100% (1/1)	--	0% (0/1)	Inpatient duration
Kuroki et al., [3]	1	100% (1/1)	--	0% (0/1)	7 months
Tanaka et al., [24]	1	100% (1/1)	0% (0/1)	0% (0/1)	2 days
Park et al., [25]	1	100% (1/1)	--	0% (0/1)	1 month
Stavridis et al., [9]	1	100% (1/1)	0% (0/1)	0% (0/1)	8 months
Allen et al., [26]	1	100% (1/1)	0% (0/1)	0% (0/1)	20 months
Li et all, [4]	1	100% (1/1)	0% (0/1)	0% (0/1)	Inpatient duration
Espada et al., [27]	1	100% (1/1)	0% (0/1)	0% (0/1)	Inpatient duration
Greco et al., [5]	1	100% (1/1)	0% (0/1)	0% (0/1)	1 year
Shibata et al., [14]	1	100% (1/1)	--	--	--
Kaza et al., [28]	1	100% (1/1)	0% (0/1)	0% (0/1)	Inpatient duration
Kudo et al., [2]	1	100% (1/1)	0% (0/1)	0% (0/1)	6 months
Irie et al., [29]	1	--	--	--	--
Misumi et al., [30]	1	100% (1/1)	0% (0/1)	--	--
Le Guyader et al. [31]	1	100% (1/1)	0% (0/1)	0% (0/1)	6 years
Kaneko et al., [32]	1	100% (1/1)	100% (1/1) (Transient AF)	0% (0/1)	Inpatient duration
Walkes et al., [33]	1	100% (1/1)	0% (0/1)	0% (0/1)	8 months
Je et al., [34]	1	100% (1/1)	0% (0/1)	0% (0/1)	Inpatient duration
Modi et al., [6]	1	100% (1/1)	0% (0/1)	0% (0/1)	2 months
Tarcan et al., [35]	1	100% (1/1)	0% (0/1)	0% (0/1)	3 months
Toeg et al., [8]	1	100% (1/1)	0% (0/1)	0% (0/1)	1 year
Akagi et al., [7]	1	100% (1/1)	0% (0/1)	0% (0/1)	Inpatient duration
Ariyoshi et al., [36]	1	--	--	--	--
Schröder et al., [37]	1	100% (1/1)	--	--	--
Nijmeh et al., [10]	1	100% (1/1)	0% (0/1)	0% (0/1)	Inpatient duration
Bauer et al., [1]	1	--	0% (0/1)	--	--
Overall		100% (50/50)	2.2% (1/46)	0% (0/47)	

The most common type of endoscopic device used was rigid endoscope [1, 5, 8, 12–14, 19, 22, 23, 32, 33, 36, 37], reported in 13 studies. This was followed by semi-rigid endoscopes in four [7, 9, 21, 29], flexible endoscopes in three [2, 3, 30] and mixture of rigid and flexible endoscopes in one study [11]. In 13 studies, the type of endoscope used was not specified [4, 6, 10, 15, 20, 24–28, 31, 34, 35]. Other adjunct endoscopic instruments such as forceps, graspers, scissors, suckers or retractors were used in 13 studies [2, 3, 12, 13, 15, 19, 21, 25, 30, 32, 34, 35, 37]. In the remaining it was not clear whether open surgical or endoscopic instruments were used. The pre-operative imaging was pre-dominantly echocardiography, noted in 31 cases [2–10, 12–15, 20–37]. In one case ventriculography was used [19] and in the remaining 22 cases the imaging method was not specified [1, 11]. In addition to pre-operative imaging with echocardiogram, in 10 cases intra-operative echocardiography was also used to ensure complete removal of the intracardiac lesions [9, 10, 12, 15, 21, 24, 26–28, 31].

### Post-operative outcomes

There were no mortalities (0/47) among 27 studies at last recorded follow-up (Table 2) [2–13, 15, 19, 21–28, 31–35]. In 12 studies, the follow-up was longer than 30-days and no mortalities were observed [2, 3, 5, 6, 8, 9, 12, 25, 26, 31, 33, 35]. The only postoperative complication observed was one case of atrial fibrillation (2.2%, 1/46). Further examination of this case revealed that the patient was in fact suffering from palpitations prior to the operation with preoperative ECG finding of multiple atrial ectopics [32]. A successful outcome, defined as complete excision of the lesion, was achieved in all cases (100%, 50/50) among 30 studies reporting this outcome [2–15, 19, 21–28, 30–35, 37].

## Discussion

The results presented here demonstrate that cardiac endoscopy has been successfully utilised with no mortality and only a single non-technique related morbidity (atrial fibrillation 1/46) in the treatment of a number of pathologies including hypertrophic cardiomyopathy, intra-cardiac tumors and intra-cardiac thrombosis.

### Case selection

Cardiac endoscopy has been successfully utilised in the treatment of a number of pathologies including hypertrophic cardiomyopathy, intra-cardiac tumours and intra-cardiac thrombosis.

In cases of hypertrophic myocardium, cardio-endoscopic techniques allow resection of tissue in deeper parts of the ventricle, whilst providing visual feedback to surgeon to facilitate controlled resection and avoid damage to nearby structures such as mitral valve, chordae, papillary muscles and septum [1]. This may lead to a more thorough resection of the septum [11]. Alternative pre-operative guidance methods such as transoesophageal echocardiography that assess extent of hypertrophic tissue before and after operation do not provide these advantages [38]. As such, the combined use of pre-operative imaging and direct visualisation with an endoscope during the procedure provides complementary information subsequently allowing the surgeon to achieve a more radical and complete resection.

Approximately 25% of primary cardiac tumours are reported to be malignant [13]. Common benign tumours include papillary fibroelastoma (PFE), myxoma and benign haemangiomas. Surgical intervention in malignant cases may not be justified due to poorer prognosis and complications associated with the procedure [13, 39]. In contrast however, the treatment of choice for benign intracardiac tumours is surgical excision. In case of papillary fibroelastomas (PFEs), the aim is to prevent life threatening coronary, systemic and cerebral emboli [26, 34] and reduce the risk of angina and sudden death through direct ostial occlusion by tumour prolapse [40]. Excision is therefore advocated even in asymptomatic patients and tends to be curative [26, 27, 31, 34, 41]. The usual approach to removing PFEs is through left atrium or aorta,[39] with preference to the aortic approach when the tumour is located in left ventricular (LV) outlet, and access through the mitral valve when the tumour is located in the LV inlet [7]. For those lesions located deeper in the ventricle, ventriculotomy may be required [7, 39, 42]. On the other hand, endoscopic methods can facilitate both direct assessment of the benign tumour and its complete removal with minimal collateral damage. This not only avoids embolisation from tumour but also has the potential to avoid recurrence [41, 43].

Similarly, cardiac myxomas and benign haemangiomas also commonly require surgical excision to prevent embolic complications or intracardiac obstruction [6, 28, 37]. Incomplete removal of myxomas is associated with high risk of recurrence [5], and as such cardio-endoscopic techniques may equally facilitate avoidance of ventriculotomy, improvement of visualisation and completeness of excision [5, 6, 10].

**Fig. 1:**
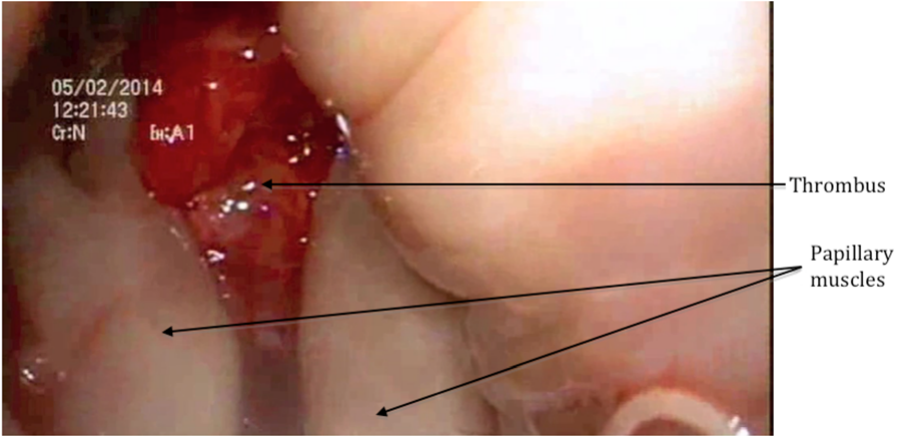
Intraoperative image taken by a cardio-endoscope through aortic valve, illustrating left ventricular thrombus

**Fig. 2:**
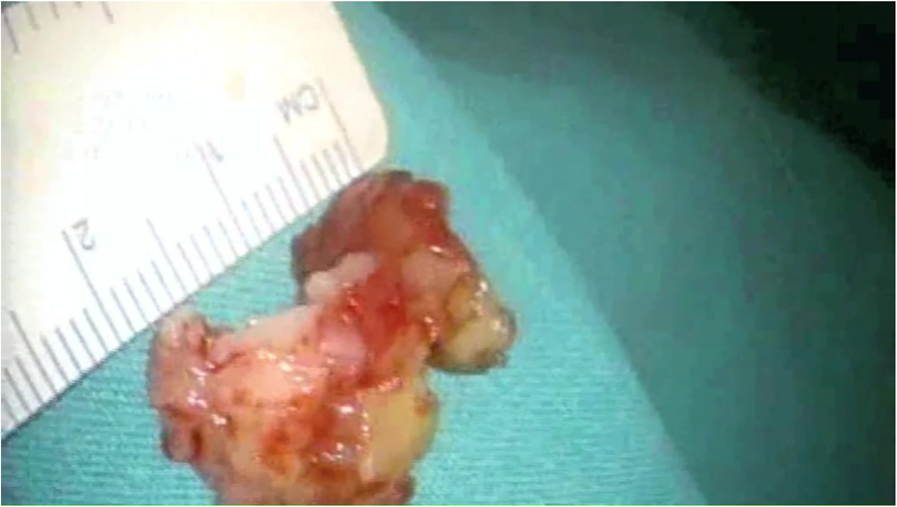
Gross appearance of the excised left ventricular thrombus

**Fig. 3:**
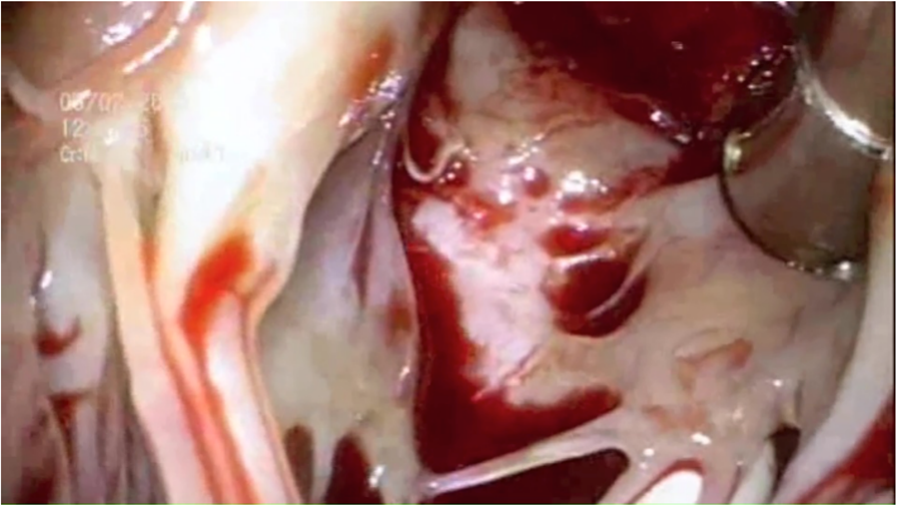
Confirmation of complete removal of the thrombus by cardio-endoscope

Left ventricular thrombus is a common complication of acute myocardial infarction[44]. The broad classification of thrombus includes mural, mobile and pedunculated thrombi, the majority of which are mural, flat and immobile with low risk of embolism [15]. Pedunculated and mobile thrombi are rarer; however, possess higher risk of coronary, cerebral and systemic embolization and surgical excision is often indicated [15, 45]. Unlike the excision of intracardiac tumours and hypertophic myocardium, the standard approach for removal of LV thrombus involves left ventriculotomy [3, 12]. In cases where thrombus is formed following an acute MI, ventriculotomy poses the added concern of closing a friable myocardium with increased risk of bleeding as well as added insult to left ventricular function [15]. Use of cardio-endoscopic methods has the advantage of preventing complications such as ventricular dysfunction, arrhythmias and left ventricular aneurysm formation and allows rapid recovery of myocardium from inflammatory process [9, 12]. An example demonstration of LV thrombus excision is shown in Figs. 1, 2 and 3.

### Minimally invasive applications

Other advantages of the cardio-endoscopic technique include potential use as an adjunct in minimally invasive and totally endoscopic cardiac surgery. In this pooled series of data a right mini thoracotomy and thoracoscopic approach were used in 5 cases. Such minimally invasive procedures are well recognized to not only improve cosmesis but also facilitate lower post-operative infection rates, shorter hospital stay and earlier return to work when compared to sternotomy [3, 6, 24, 46]. As such, the employment of cardio-endoscopic visualization with minimal access incisions for treatment of LV lesions, would be expected to produce similar benefits [6]. Furthermore, this technique also has the benefit of providing educational value to the whole surgical team including trainees, nursing staff, anaesthetists and medical students who can follow the operation on the screen [1]. This may particularly important where the assistant’s view is limited, as is the case in minimally invasive, mitral valve, and congenital procedures [11].

### Technical considerations

Rigid endoscopes were the most commonly reported device type in this pooled series of data. There have been no comments about advantages and disadvantages of using rigid versus flexible versus semi-rigid (rigid body with flexible tip) endoscopes. However, limitations with the endoscopic technique includes interference between other instruments and the endoscope and soiling of the camera with blood during the procedure, subsequently obscuring vision [11]. The latter can be addressed with an effective rinsing and sucking device [11], which needs to be developed and incorporated into endoscope. The former problem partly depends on the lesion location and instruments being used. Adjunct endoscopic instruments such as forceps, graspers, scissors, suckers or retractors were used in 13 studies. None reported problems due to interference of their surgical equipment with the endoscope.

The average cross clamp and cardio-pulmonary bypass time reported in this series of cases was 59.5 and 105.3 min respectively. There were no comparative studies for us to contrast these data with procedures performed under direct vision. Whether or not above operation times could be improved over time also remain open to question and would be one of the factors in demonstrating the learning curve involved in development of this practice.

Another point to consider is that in the majority of cases only one lesion was removed from the left ventricle. However, there were no differences in postoperative outcomes for different number of lesions resected. The sizes of the lesions from different studies were difficult to compare and collate due to differences in reporting. We believe that number and size of the lesions in the left ventricle may impact on the practicality of endoscopic resection. As such, these two variables should be taken into account in In future clinical trials.

### Limitations

There are several limitations in both study design and outcome reporting that influence the interpretation of these results. Firstly, there were no comparative studies to enable direct comparison of outcomes for cases performed under direct vision or ventriculotomy with cardio-endoscopic technique. This prevents us drawing definitive conclusions on safety and efficacy of the technique or on the mean cross-clamp and cardiopulmonary bypass times observed. Secondly, the majority of studies were case reports and only one small case series, limiting the power of detecting adverse events associated with the technique. Furthermore, inter study variations in size, nature and location of the left ventricular lesions, concomitant procedures and type of endoscopic instrument used likely to influence pooled results and should be taken into consideration when interpreting outcome of the data. Similarly, the case based nature of the majority of these studies means that there is a significant risk of publication bias that may over-represent favourable results with preference given to the reporting of successful procedures. Finally, we were unable to obtain data regarding the financial cost of the cardio-endoscopic technique in these scenarios. Further information regarding the cost-effectiveness of more widespread adoption of this technique is required.

## Conclusions

Cardio-endoscopic techniques provide a useful adjunct to the surgeon’s armamentarium in resecting pathologic tissue from the left ventricle. Resection was successful in all cases reported in this pooled series of data and thought to be associated with better visualisation of the pathological and healthy neighbouring structures.

The absence of mortality and only single morbidity in this pooled series is encouraging. Traditionally, left ventricular lesions are excised under direct vision via aorta, left atrium or ventriculotomy. Use of a cardio-endoscopic technique, avoids exposure related valvular damage and adverse effects associated with ventriculotomy. Other advantages include added potential for these devices as an adjunct to minimally invasive cardiac surgery and potential to improve education during intra-cardiac procedures.

The data included in this pooled analysis constitutes a heterogonous patient population from case reports and a single non-randomised case series. In order to provide a more quantitative analysis, further adequately powered phase IV clinical trials are needed to compare cardio-endoscopic devices against procedures performed under direct vision. Careful patient selection, taking into account factors such as size, nature and location of left ventricular lesions and choice of endoscopic instruments should be pre-determined. The results should detail both short and long-term morbidity and mortality outcomes, particularly focusing on combined major cardiac and cerebrovascular events.

## Additional file

Additional file 1: Table S1. Synopsis of studies. (DOCX 35 kb)

## Abbreviations

AF: atrial fibrillation
CABG: coronary artery bypass grafting
IL: interleukin
LV: left ventricle
MACCE: major cardiac and cerebrovascular events
MI: myocardial infarction
PFEs: papillary fibroelastomas
