# Supplementary material for: A systematic review of left ventricular cardio-endoscopic surgery
Source: J Cardiothorac Surg. 2017 May 25;12:41. doi: 10.1186/s13019-017-0599-z (PMC5445499; doi:10.1186/s13019-017-0599-z)
Supplement: Additional file 1: Table S1. — Synopsis of studies. (DOCX 35 kb) [file 13019_2017_599_MOESM1_ESM.docx]

**Table S1: Synopsis of studies**

| **Study** | **Study Type** | **n** | **Age** | **Gender**  **(M: F)** | **Indication** | **Histopathology** | **Size of lesion (cm)** | **Location of lesion in LV** | **Concomitant procedure** | **Incision** | **Entry site** | **CX** | **CPB** | | **Endoscopic instrument(E)/ Adjuncts(A)** |
| --- | --- | --- | --- | --- | --- | --- | --- | --- | --- | --- | --- | --- | --- | --- | --- |
|  |  |  |  |  |  |  |  |  |  |  |  | **Time (min)** | | |  |
| Duarte et al, 1997 [19] | Case study | 1 | 58 | M | Thrombus | -- | 2 X 2.5 | Apex | No | Median Sternotomy | Aortotomy | 31 | | 45 | E: Video thoracoscope (10mm; Karl-Storz, Germany)  A: Thoracoscopic alligator dissecting forceps (4mm; Karl-Storz, Germany) |
| Mazza et al, 1998 [12] | Case study | 1 | 17 | F | Thrombus | -- | 3 | Apex | No | Median Sternotomy | Aortotomy | 81 | | -- | E: Endoscope  (4mm, 30 degree; Smith and Nephew, Dyonics, Inc, Andover, MA)  A: 2.7mm endoscopic grasping forceps |
| Tsukube et al, 1999 [15] | Case study | 1 | 59 | M | Thrombus | -- | 2.0 X 2.0 | Body | No | Median Sternotomy | Aortotomy | -- | | -- | E: Videothoracoscope  A:Videothoracoscopic equipment |
| Reuthebuch et al, 1999 [11] | Case series | 15 | -- | -- | Hypertrophic tissue | -- |  | Body | No | Median Sternotomy | Aortotomy | -- | | -- | E: Cardioscope  (Rigid: 5mm: 0, 30, 70 degree angles; Flexible: 0 degree; Karl Storz, Tuttlingen, Germany)  A: Elongated forceps, scissors or scalpels. |
|  |  | 4 | -- | -- | Thrombus | -- | -- | -- | No | Median Sternotomy | Aortotomy | -- | | -- |  |
|  |  | 2 | -- | -- | Tumour | Papillary fibroelastoma | -- | -- | No | Median Sternotomy | Aortotomy | -- | | -- |  |
| Junemann-Ramirez et al, 2005[13] | Case study | 1 | 60 | M | Thrombus | -- | 2 X 3 | Body | No | Median Sternotomy | Aortotomy | -- | | -- | E: Thoracocscope  (Rigid, 10 mm, 0 degree angles; Storz)  A: Thoracoscopic equipment |
| Oumeiri et al, 2007 [20] | Case study | 1 | 52 | M | Thrombus | -- | -- | Apex | -- | -- | -- | -- | | -- | -- |
| Kawamoto et al, 2008 [21] | Case study | 1 | 44 | M | Thrombus | -- | 3.5 X 1.8 | Body | No | Median Sternotomy | Aortotomy | -- | | -- | E: Endoscope  (10 mm, 30 degree; Olympus, Tokyo, Japan)  A: Endoscopic grasper |
| Porcu et al, 2008[22] | Case study | 1 | 31 | M | Thrombus | -- | 3.5 X 1.5 | Body | No | Median Sternotomy | Left atriotomy | -- | | -- | E: Endoscope  (Karl Storz, Optique Hopkins II O◦ type 26003 AA, Tuttlingen, Germany)  A: Forceps |
| Kikuchi et al, 2009 [23] | Case study | 1 | 59 | M | Thrombus | -- | 1.6, 1.8 | Apex | Yes | Median Sternotomy | Aortotomy | 60 | | -- | E: Endoscope (Rigid, 5mm)  A: Forceps |
| Kuroki et al, 2012 [3] | Case study | 1 | 58 | F | Thrombus | -- | 2 | Apex | No | Right mini thoracotomy | Left atriotomy | 23 | | -- | E: Thoracoscope (Flexible)  A: Endoscopic grasping forceps |
| Tanaka et al, 2013 [24] | Case study | 1 | 40 | M | Thrombus | -- | 1.4 X 3.5 | Body | No | Right mini thoracotomy | Left atriotomy | 45 | | 82 | A: Video-assisted thoracoscopy 3D Endoscope (ShinkoOptical Co.,Ltd., Tokyo, Japan)  A: ValvaGate DeBakey Grasper (GEISTER Medizintechnik GmbH, Tuttlingen, Germany) |
| Park et al, 2014 [25] | Case study | 1 | 63 | M | Thrombus | -- | 1.75 X 1.48 | Body | Yes | Median Sternotomy | Aortotomy | 218 | | 355 | E: Endoscope (10-mm)  A: Endoscopic forceps |
| Stavridis et al, 2015 [9] | Case study | 1 | 56 | M | Thrombus | -- | 2.5 | Body | Yes | Median Sternotomy | Aortotomy | -- | | -- | E: Videolaparoscope (flexible tip; Olympus TM LTF-VP)  A:Long surgical forceps |
| Allen et al, 1996 [26] | Case study | 1 | 57 | M | Tumour | Papillary fibroelastoma | 1.5 X 1.5 | Papillary muscles | No | Median Sternotomy | Aortotomy | -- | | -- | E: Videothoracosope |
| Li et all, 1996 [4] | Case study | 1 | 20 | F | Tumour | Myxoma | 2.1 X 1.9 | Body | No | -- | Aortotomy | -- | | -- | E: Cardioscope |
| Espada et al, 1997 [27] | Case study | 1 | 62 | M | Tumour | Papillary fibroelastoma | 3 X 2 X 0.5 | Body | Yes | -- | Left atriotomy | -- | | -- | E: Video-assisted thoracoscope (30-degree angle with three chip camera; Stryker, Santa Clara, CA) |
| Greco et al, 1999 [5] | Case study | 1 | 23 | M | Tumour | Myxoma | 2 X 2 | Apex | No | Median Sternotomy | Aortotomy | -- | | -- | E: Thoracoscope (10-mm, 0 degree angle, Storz) |
| Shibata et al, 1999 [14] | Case study | 1 | 26 | F | Tumour | Papillary fibroelastoma | -- | Body | No | Median Sternotomy | Aortotomy | -- | | -- | E: Optical telescope (Rigid, 5-mm diameter, 30 degree angle; Karl Storz, Tubingen, Germany)  A: Grasping forceps (Nishihata, Nagashima, Tokyo, Japan)  Cavitron Ultrasonic Surgical Aspirator (Valleylab, Boulder, CO,USA) |
| Kaza et al, 2002[28] | Case study | 1 | 56 | M | Tumour | Benign hemangioma | 1 | Body | No | Median Sternotomy | Aortotomy | -- | | -- | E: Video-assisted cardioscope  (10 mm, 30-degree)  A:Scalpel |
| Kudo et al, 2004 [2] | Case study | 1 | 82 | F | Tumour | Papillary fibroelastoma | 2 | Body | No | Median Sternotomy | Left atriotomy | -- | | -- | E: Endoscope  (XQ240, Olympus, Co., Tokyo, Japan)  A: Snare strangulation (Erbotom ICC200, Tubingen, Germany)  Biopsy forceps. |
| Irie et al, 2004 [29] | Case study | 1 | 59 | M | Tumour | Papillary fibroelastoma | 5 X 6 | Body | No | -- | Aortotomy | -- | | -- | E: Video-assisted thoracoscope  (5 mm diameter; Olympus, Japan) |
| Misumi et al, 2005[30] | Case study | 1 | 63 | M | Tumour | Papillary fibroelastoma | 2 | Body | No | Median Sternotomy | Aortotomy | -- | | -- | E: Endoscope  (Flexible, XQ240; Olympus, Tokyo, Japan)  A: Snaring  (ERB ICC200, Tokyo, Japan)  Biopsy forceps. |
| Le Guyader et al 2006 [31] | Case study | 1 | 55 | M | Tumour | Papillary fibroelastoma | 1 | Chords | No | -- | Aortotomy | -- | | -- | E: Thoracoscope  A: Thoracoscopic instruments |
| Kaneko et al, 2006[32] | Case study | 1 | 81 | F | Tumour | Papillary fibroelastoma | 1.1 X 1.2 | Apex | No | Median Sternotomy | Left atriotomy | 34 | | 79 | E: Videoscope  (30-degree, rigid)  A: Endoscopic sucker and scissors |
| Walkes et al, 2007 [33] | Case study | 1 | 61 | M | Tumour | Papillary fibroelastoma | -- | Apex | No | Median Sternotomy | Aortotomy | -- | | -- | E: Thoracoscope  (Rigid, 2-mm) |
| Je et al, 2008 [34] | Case study | 1 | 39 | M | Tumour | Papillary fibroelastoma | 1.5 X 1.0 | Papillary muscles | No | Right mini thoracotomy | Left atriotomy | 24 | | 57 | E: Voice-activated robotic camera  (AESOP 3000)  A: Chitwood hand-held left atrial retractor |
| Modi et al, 2009 [6] | Case study | 1 | 65 | M | Tumour | Myxoma | 1.2 X 0.9 | Body | No | Right mini thoracotomy | Aortotomy | -- | | 66 | E: Thoracoscope |
| Tarcan et al, 2011 [35] | Case study | 1 | 28 | M | Tumour | Myxoma | 1.2 X 1 | Chords | No | Right Thoracoscopy | Left atriotomy | 57 | | 121 | E: Two dimensional camera  (Storz, Tuttlingen, Germany)  A: Endoscopic instruments |
| Toeg et al, 2012 [8] | Case study | 1 | 68 | M | Tumour | Papillary fibroelastoma | 1.1 X 1.6 | Apex | No | Median Sternotomy | Aortotomy | 22 | | 37 | E: Mediastinoscope  (Rigid; Storz Instruments, Tuttlingen, Germany)  A: Biopsy forceps |
| Akagi et al, 2013 [7] | Case study | 1 | 74 | F | Tumour | Papillary fibroelastoma | 1.9 X 1.5 | Papillary muscles | Yes | Median Sternotomy | Left atriotomy | -- | | -- | E: Thoracoscope  (5-mm ; Endoeye Flex LTFS190-5; Olympus Optical, Tokyo, Japan) |
| Ariyoshi et al, 2013 [36] | Case study | 1 | 81 | F | Tumour | Papillary fibroelastoma | 1.4 × 1.1 | Apex | No | Median Sternotomy | Left atriotomy | -- | | -- | E: Videoscope (Rigid) |
| Schröder et al, 2013 [37] | Case study | 1 | 57 | F | Tumour | Myxoma | 3.6 | Body | No | Median Sternotomy | Aortotomy | -- | | -- | E: Endoscope  (Rigid, 5-mm , 30 degree angle)  A: Ring forceps, 5-mm grasper and endoshears |
| Nijmeh et al, 2013 [10] | Case study | 1 | 31 | F | Tumour | Myxoma | 3 | Body | No | -- | Left atriotomy | -- | | -- | E: Cardioscope |
| Bauer et al, 1997 [1] | Case study | 1 | -- | -- | Hypertrophic and fibrous tissue | -- |  | Body | No | Median Sternotomy | Aortotomy | -- | | -- | E: Cardioscope  (Rigid, Storz Instruments, Tuttlingen, Germany)  A: Scalpel, Retractor, Rongeur |
